# 20-Hydroxyecdysone Primes Innate Immune Responses That Limit Bacterial and Malarial Parasite Survival in Anopheles gambiae

**DOI:** 10.1128/mSphere.00983-19

**Published:** 2020-04-15

**Authors:** Rebekah A. Reynolds, Hyeogsun Kwon, Ryan C. Smith

**Affiliations:** a Department of Entomology, Iowa State University, Ames, Iowa, USA; Johns Hopkins Bloomberg School of Public Health

**Keywords:** 20-hydroxyecdysone, *Plasmodium*, bacteria, hormone, immune priming, innate immunity, mosquito

## Abstract

Blood feeding is an integral behavior of mosquitoes to acquire nutritional resources needed for reproduction. This requirement also enables mosquitoes to serve as efficient vectors to acquire and potentially transmit a multitude of mosquito-borne diseases, most notably malaria. Recent studies suggest that mosquito immunity is stimulated following a blood meal, independent of infection status. Since blood feeding promotes production of the hormone 20-hydroxyecdysone (20E), we hypothesized that 20E plays an important role in priming the immune response for pathogen challenge. Here, we examine the immunological effects of priming Anopheles gambiae with 20E prior to pathogen infection, demonstrating a significant reduction in bacteria and Plasmodium berghei survival in the mosquito host. Transcriptome sequencing (RNA-seq) analysis following 20E treatment identifies several known 20E-regulated genes, as well as several immune genes with previously reported function in antipathogen defense. Together, these data demonstrate that 20E influences cellular immune function and antipathogen immunity following mosquito blood feeding, arguing the importance of hormones in the regulation of mosquito innate immune function.

**IMPORTANCE** Blood feeding is required to provide nutrients for mosquito egg production and serves as a mechanism to acquire and transmit pathogens. Shortly after a blood meal is taken, there is a peak in the production of 20-hydroxyecdysone (20E), a mosquito hormone that initiates physiological changes, including yolk protein production and mating refractoriness. Here, we examine additional roles of 20E in the regulation of mosquito immunity, demonstrating that priming the immune system with 20E increases mosquito resistance to pathogens. We identify differentially expressed genes in response to 20E treatment, including several involved in innate immune function as well as lipid metabolism and transport. Together, these data argue that 20E stimulates mosquito cellular immune function and innate immunity shortly after blood feeding.

## Abstract (precis)

Blood feeding is required to provide nutrients for mosquito egg production and serves as a mechanism to acquire and transmit pathogens. Shortly after a blood meal is taken, there is a peak in the production of 20-hydroxyecdysone (20E), a mosquito hormone that initiates physiological changes, including yolk protein production and mating refractoriness. Here, we examine additional roles of 20E in the regulation of mosquito immunity, demonstrating that priming the immune system with 20E increases mosquito resistance to pathogens. We identify differentially expressed genes in response to 20E treatment, including several involved in innate immune function as well as lipid metabolism and transport. Together, these data argue that 20E stimulates mosquito cellular immune function and innate immunity shortly after blood feeding.

## INTRODUCTION

Blood-feeding behavior evolved in mosquitoes to provide nutritional resources required for egg development. While providing benefits for reproduction, blood feeding also exposes mosquitoes to a myriad of blood-borne pathogens that can ultimately be transmitted to a new host through an additional blood meal. For this reason, mosquitoes are arguably the deadliest animals on the planet, causing hundreds of millions of infections and over 500,000 deaths every year. Of mosquito-borne diseases, malaria continues to be the most deadly, with more than 400,000 people dying annually from *Plasmodium* parasite infection transmitted by female *Anopheles* mosquitoes (1).

Following a blood meal, signals initiated in the brain produce ovarian ecdysteroidogenic hormone (OEH) and insulin-like peptides (ILPs), which trigger ecdysone production by the ovaries (2–4). Ecdysone is then converted into 20-hydroxyecdysone (20E) by hydroxylation in the fat body, stimulating the production of yolk protein precursors (YPPs) in a process known as vitellogenesis (2, 5–7). While the influence of 20E in initiating vitellogenesis has been examined predominantly in Aedes aegypti (2–7), evidence suggests that these signals are conserved in Anopheles gambiae (8, 9). Reaching peak levels approximately 18 to 24 h after blood feeding (9, 10), the production of 20E in *Anopheles* also coincides with *Plasmodium* ookinete invasion of the midgut epithelium (11, 12). Although the influence of 20E is well established across insect systems with respect to development (13, 14), mating (15–17), reproduction (7, 18, 19), and vectorial capacity (20, 21), only a few studies have examined the influence of 20E on innate immune signaling in insects (22–28).

In *Drosophila*, evidence suggests that 20E mediates cellular immunity (26) and regulates a subset of antimicrobial peptides (AMPs) involved in antibacterial defense through peptidoglycan recognition protein LC (PGRP-LC)-dependent and PGRP-LC-independent mechanisms of the immunodeficiency (IMD) pathway activation (23, 24). However, few studies have examined the influence of 20E on mosquito innate immunity, thus far implicating 20E in the regulation of prophenoloxidase (PPO) expression (27) and leucine-rich repeat immune protein 9 (LRIM9) (28), both of which are known effectors of anti-*Plasmodium* immunity (28, 29). Additional studies have suggested that 20E may also mediate mosquito immune cell activation following blood feeding (30–32), implying that 20E is an important determinant of *An. gambiae* cellular immune function. Despite these previous associations, we have little understanding of how 20E signaling influences gene expression and immune function in *An. gambiae*.

Recent studies have demonstrated that 20E signaling is intricately tied to *Plasmodium* development in the mosquito host (20, 21, 33). Genetic approaches to ablate ovary development and impair 20E endogenous production imply that ecdysone signaling is required for malaria parasite development via the production of mosquito host-derived lipids (21) and the suppression of mosquito complement function through the production of vitellogenin (33). However, these studies are contrasted by the topical application of a 20E agonist above physiological 20E levels prior to *Plasmodium* infection, which significantly reduces parasite infection (20) and suggests that 20E signaling stimulates innate immune function similar to other insect systems (22–26). Together, these data suggest that 20E signaling has dual roles, enabling the establishment of parasite infection while boosting anti-*Plasmodium* responses that limit parasite survival, likely through distinct mechanisms.

To better understand how 20E acts on mosquito immune function, here we examine the influence of 20E on immune priming prior to pathogen challenge. We demonstrate that 20E increases phagocytic activity and that 20E priming reduces bacteria and *Plasmodium* survival. Through transcriptome sequencing (RNA-seq) analysis, we demonstrate that 20E application induces known components of 20E signaling as well as several immune genes implicated in mosquito immunity, suggesting that 20E primes mosquito innate immunity for pathogen challenge.

## RESULTS

### Blood feeding and 20E injection increase phagocytic activity.

**FIG 1:**
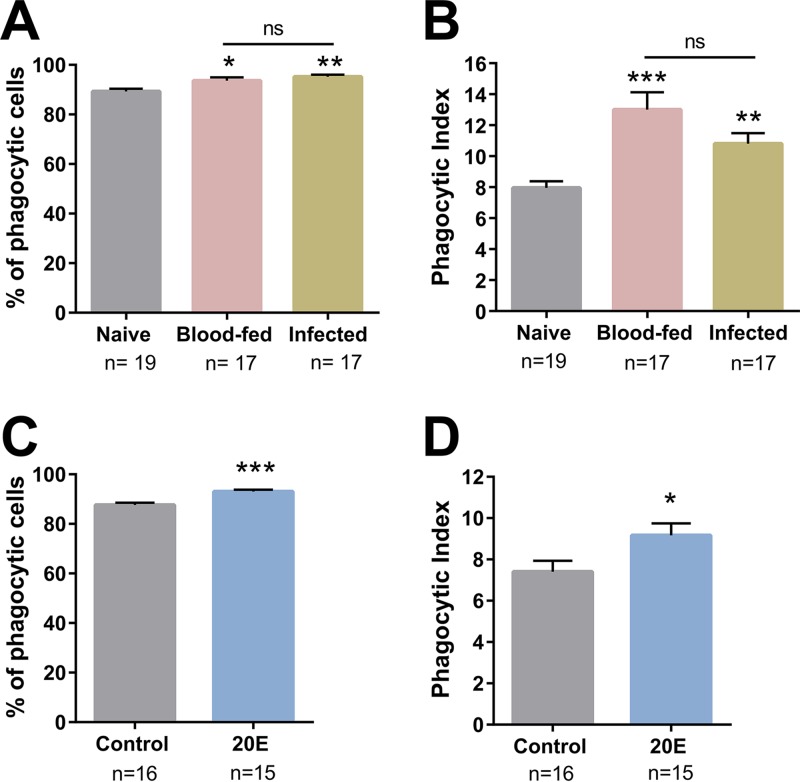
Phagocytic activity increases following blood feeding and 20E injection. (A and B) Phagocytosis assays were performed in adult female *An. gambiae* mosquitoes under naive, blood-fed, or P. berghei-infected conditions approximately 24 h postfeeding. Perfused hemocytes from each condition were evaluated by the percentage of phagocytic cells (A) and the phagocytic index (number of beads per cell) (B). (C and D) Similar experiments were performed following 20E priming, where the influence of 20E was evaluated by the percentage of phagocytic cells (C) and the phagocytic index (D). Three or more independent experiments were performed for each treatment. Data were analyzed by Kruskal-Wallis with a Dunn’s posttest (A and B) or a Mann-Whitney test (C and D) using GraphPad Prism 6.0. *n*, number of mosquitoes examined for each condition. Asterisks denote significance: *, *P < *0.05; **, *P < *0.01; ***, *P < *0.001). ns, not significant.

To determine the effects of 20E on mosquito immune function, we first explored whether blood feeding and 20E influenced mosquito cellular immunity. Immune cells, known as hemocytes, serve as primary immune sentinels that recognize and destroy invading pathogens by phagocytosis or through the production of humoral immune factors (34, 35). To examine how blood feeding and 20E influence these phagocytic properties, we injected fluorescent beads to evaluate phagocytosis under different physiological conditions, as previously described (29, 36). Similar to the findings of Kwon and Smith (29), we demonstrate that blood feeding, independent of pathogen challenge, significantly increased the percentage of phagocytic cells (Fig. 1A) and phagocytic activity (Fig. 1B). We therefore hypothesized that the increase in phagocytic activity might be influenced in part by the increased levels of 20E post-blood feeding. To address this question, we injected 20E into mosquitoes prior to challenge with fluorescent beads. Similarly, we observed a significant increase in the proportion of phagocytic cells (Fig. 1C) and phagocytic activity (Fig. 1D) 24 h after 20E injection, suggesting that 20E activates mosquito cellular immunity.

### Blood feeding and 20E limit bacterial infection.

**FIG 2:**
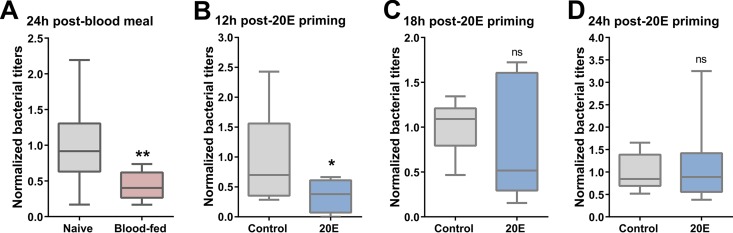
Blood feeding and 20E priming reduce E. coli survival. (A to D) The effects of blood feeding and 20E priming on bacterial titers were examined through bacterial challenge experiments with E. coli. Mosquitoes were challenged with bacteria ∼24 h post-blood meal (A), 12 h post-20E priming (B), 18 h post-20E priming (C), or 24 h post-20E priming (D). For each experimental condition, hemolymph bacterial titers were examined 24 h later by perfusion. Pooled hemolymph from five mosquitoes was plated in duplicate on LB-kanamycin plates, and the bacterial colonies were counted to determine the titers. Data were normalized from three or more independent experiments and visualized using box plots (whiskers show minimum/maximum values, lines denotes median values) and evaluated by Mann-Whitney analysis using GraphPad Prism 6.0 to determine the significance. *, *P < *0.05; **, *P < *0.01; ns, not significant.

Based on our observations that blood feeding and 20E increase phagocytic activity, we next looked to determine the role of blood feeding and 20E priming on bacterial challenge. To approach this question, we challenged naive and blood-fed mosquitoes (∼24 h postfeeding) with Escherichia coli, and bacterial titers were evaluated 18 to 24 h later from perfused hemolymph. Comparisons of naive and blood-fed mosquitoes revealed a significant reduction in bacterial numbers in blood-fed mosquitoes when E. coli titers were examined at 24 h (Fig. 2A). Since 20E is produced following a blood meal, we speculated that 20E signaling increased immune function and subsequently reduced E. coli survival. This was examined by priming mosquitoes with 20E and then challenging as done previously with E. coli at 12, 18, or 24 h post-20E priming. We found a significant reduction in E. coli titers when challenged 12 h post-20E priming (Fig. 2B), yet the effects of 20E priming were abrogated when challenged at 18 (Fig. 2C) or 24 (Fig. 2D) h post-20E priming. Interestingly, this peak effect on antibacterial immunity occurs ∼12 h post-20E priming. This corresponds to the approximate timing of peak 20E production in the mosquito host, where 20E is induced ∼12 h post-blood meal and peaks between 18 and 24 h post-blood feeding (37).

### 20E priming limits *Plasmodium* survival.

**FIG 3:**
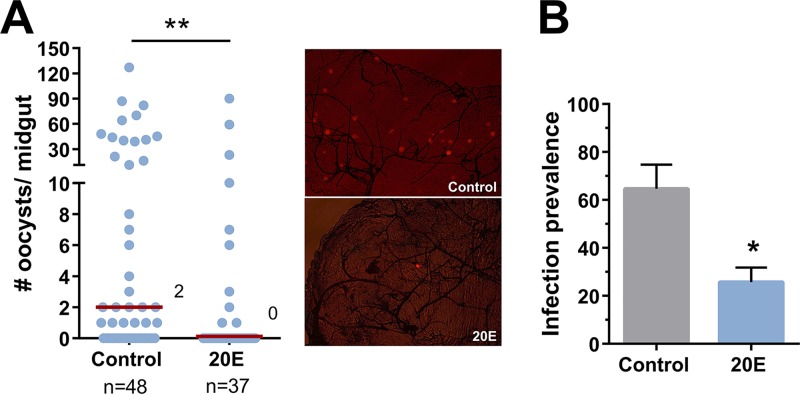
20E priming significantly reduces *Plasmodium* survival. (A) Adult female mosquitoes were injected with 10% EtOH in 1× PBS (control) or 20E and then challenged with a P. berghei-infected mouse 24 h later. Eight days postinfection, *Plasmodium* oocyst numbers were evaluated by fluorescence, as shown in representative images for each condition. Data were pooled from four independent experiments. Median oocyst numbers from both treatments are represented by a red line. (B) The percentages of mosquitoes containing at least one oocyst (prevalence of infection) were combined from each of the four independent experiments. Mann-Whitney analysis was used to determine significance using GraphPad Prism 6.0. *, *P < *0.05; **, *P < *0.01. *n*, number of mosquitoes examined per treatment.

Since 20E influences phagocytosis and antibacterial immunity (Fig. 1 and 2), we wanted to explore whether 20E priming similarly influences malaria parasite numbers in the mosquito host. To examine this question, we primed mosquitoes with 20E and then challenged them with Plasmodium berghei. We found that the injection of 20E 24 h before *Plasmodium* infection significantly reduced parasite survival (Fig. 3A) and the prevalence of infection (Fig. 3B), suggesting that 20E initiates anti-*Plasmodium* immune responses that prime the mosquito host. No differences in feeding efficiency or in the size of the blood meal were observed following 20E injection.

### 20E regulation of mosquito physiology and immunity.

**FIG 4:**
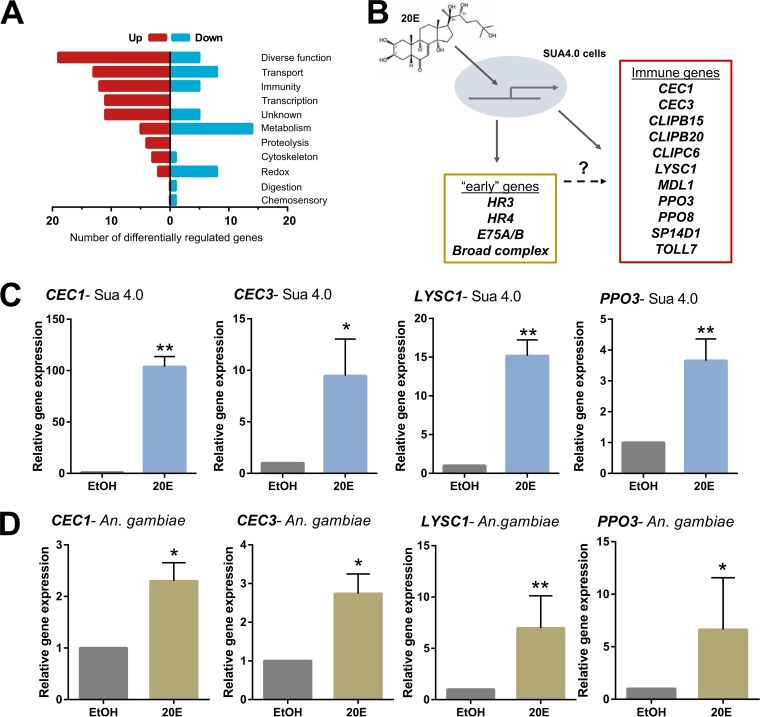
RNA sequencing identifies immune genes stimulated by 20E application. (A) RNA-seq analyses of 20E-treated Sua 4.0 cells identified 128 differentially regulated genes (80 upregulated, 48 downregulated) grouped by gene ontology. (B) Included among these genes are a number of previously described “early” genes under 20E regulation, as well as multiple genes involved in innate immunity. (C and D) Four of these immune genes (*CEC1*, *CEC3*, *LYSC1*, *PPO3*) were examined to validate the effects of 20E in either Sua 4.0 cells (C) or *in vivo* in whole female *An. gambiae* samples (D) in comparison to control (EtOH) treatments. Statistical significance was determined by Mann-Whitney analysis (*, *P < *0.05; **, *P < *0.01) from four independent biological samples.

To better understand how 20E primes immune responses to bacteria and malaria parasites, we performed RNA-seq analysis to identify genes responsive to 20E treatment. Similar to studies in *Drosophila* that examined the effects of 20E on S2 cells (24), we examined the response to 20E treatment in *An. gambiae* Sua 4.0 cells to simplify the multiple tissues and time points that might complicate expression analysis *in vivo*. Our analysis identified 128 differentially regulated genes, including 80 upregulated genes and 48 downregulated genes (see Table S3 in the supplemental material). Gene ontology analysis demonstrated that 20E application upregulates transcripts associated with transport, immunity, and transcription while downregulating transcripts with predicted function in metabolism and redox metabolism (Fig. 4A). This includes the upregulation of several previously described 20E-induced “early” genes, *HR3*, *HR4*, *E75A/B*, and multiple broad complex isoforms (Fig. 4B; Table S3), implicated in canonical 20E signaling (38).

The RNA-seq data also provide insight into many of the physiological processes that accompany mosquito blood feeding that may be under hormonal regulation. 20E treatment significantly increased the expression of allantoinase (Table S3; Fig. S1), a key enzyme in urea synthesis (39, 40), and collagen IV, a major component of the basal lamina (41). Both genes have previously been induced after blood feeding (40, 41), supporting that 20E regulation may drive these patterns in gene expression. Several genes involved in lipid metabolism and transport also displayed differential expression following 20E treatment, including the increased expression of fatty acid coenzyme A (CoA) ligase and fatty acid amide hydrolase involved in lipid synthesis and the reduced expression of apolipophorin III (Table S3). This supports that 20E mediates lipid metabolism in *An. gambiae*, similar to findings in other mosquito species (42).

10.1128/mSphere.00983-19.1 FIG S1 Validation of RNA-seq data in Sua 4.0 cells and whole mosquito samples. (A) Differentially expressed genes identified in our RNA-seq analysis were validated independently in *An. gambiae* Sua 4.0 mosquito cells and whole *An. gambiae* female mosquitoes (G3 strain) by qRT-PCR. Expression data are displayed as the mean log_2_ fold change (±SEM) from at least three independent experiments. (B and C) The RNA-seq data were correlated with qRT-PCR expression values using either Sua 4.0 cell samples (B) or whole mosquitoes (C) to validate the differential gene expression. Data were analyzed using linear regression to determine significance. Download FIG S1, TIF file, 2.8 MB. Copyright © 2020 Reynolds et al. 2020 Reynolds et al. This content is distributed under the terms of the Creative Commons Attribution 4.0 International license.

In addition, 20E application upregulated 12 genes associated with mosquito immune function (Fig. 4B; Table S1). *PPO3* is a known *Plasmodium* antagonist (29), while three other genes, *LYSC1*, *CLIPB15*, and *MDL1*, are antagonists against both bacteria and malaria parasites (43–47). The antimicrobial peptides CEC1 and CEC3 (Fig. 4B; Table S1) are widely implicated in general antipathogen effects for both bacteria and parasites (48–50), and while the exact function of CLIPC6, CLIPB20, and SP14D1 remain undetermined, researchers previously implicated these members of the CLIP protease family in mosquito immune signaling (51, 52). Together, this suggests that 20E treatment affects mosquito immunity through the regulation of several previously characterized immune genes.

10.1128/mSphere.00983-19.3 TABLE S1 Primers used for qRT-PCR analysis. Download Table S1, PDF file, 0.04 MB. Copyright © 2020 Reynolds et al. 2020 Reynolds et al. This content is distributed under the terms of the Creative Commons Attribution 4.0 International license.

We validated our RNA-seq results using a subset of differentially regulated genes *in vitro* (Fig. 4C; Fig. S1) and *in vivo* using whole mosquito samples following 20E injection (Fig. 4D; Fig. S1) by quantitative reverse transcription PCR (qRT-PCR). Gene expression significantly correlated with both *in vitro* and *in vivo* samples (Fig. S1), although gene expression results more closely matched the *in vitro* samples, as expected. These differences are exemplified by differences in the expression of several genes between mosquito cell lines and whole mosquito samples, where in some cases, 20E significantly influenced expression only *in vitro* (Fig. S1). However, we cannot exclude that 20E may significantly influence the expression of these transcripts in specific mosquito tissues.

Based on the influence of 20E priming in limiting pathogen survival (Fig. 2 and 3) and the number of differentially expressed immune genes after 20E exposure (Fig. 4; Table S3), we wanted to address the ability of these immune genes to mediate the antipathogen phenotypes produced by 20E priming. Several of the immune genes responsive to 20E treatment (Fig. 4) have been previously described (43–51), providing strong support that one or more of these immune genes are responsible for the effects of 20E-mediated immunity. We confirmed the role of one of these genes, *CEC3*, encoding an antimicrobial peptide (AMP) responsive to 20E (Fig. 4), in bacterial and parasite survival (Fig. S2). Following *cec3* silencing, E. coli titers significantly increased under naive conditions (Fig. S2) similar to previous studies (48), yet these effects were abrogated in blood-fed mosquitoes (Fig. S2). This suggests that the effects of *cec3* silencing are overcome by the large number of immune components induced by blood feeding that mask the role of CEC3 in antibacterial immunity. *cec3* silencing also resulted in a significant increase in P. berghei numbers (Fig. S2), suggesting that CEC3 contributes to anti-*Plasmodium* immunity, as described in previous studies (48–50). Together, these experiments demonstrate that 20E-mediated regulation of *CEC3*, as well as other previously described genes, provides generalized antipathogen effects that mediate both bacterial and malaria parasite survival.

10.1128/mSphere.00983-19.2 FIG S2 Cecropin 3 (*cec3*) silencing increases bacterial and P. berghei infection. (A) The efficiency of *cec3* silencing was examined in whole mosquitoes by qRT-PCR by comparing *cec3* transcripts in dsGFP (control) or dsCec3 samples. Statistical analysis was performed using Student’s *t* test with four independent experiments. (C) To determine the role of *cec3* in response to bacterial challenge, bacterial titers were examined in individual mosquitoes following the injection of dsGFP (control) or dsCec3 under naïve and blood-fed conditions. More than 30 mosquitoes were examined for each experimental condition, with data analyzed using a Mann-Whitney test to determine significance. (C) In addition, the effects of *cec3* silencing were examined on P. berghei oocyst numbers 8 days postinfection and analyzed using a Mann-Whitney test. The percentages of mosquitoes containing at least one oocyst (prevalence of infection) were combined from each of the three independent experiments and analyzed using a Mann-Whitney test. For each experiment, asterisks denote significance: *, *P < *0.05; ***, *P < *0.001. ns, not significant; *n*, number of mosquitoes examined per treatment. Download FIG S2, TIF file, 1.6 MB. Copyright © 2020 Reynolds et al. 2020 Reynolds et al. This content is distributed under the terms of the Creative Commons Attribution 4.0 International license.

## DISCUSSION

Blood feeding is an essential behavior for *An. gambiae* and other hematophagous insects to acquire nutrient resources for egg production. This requires the synthesis of yolk protein precursors in a process known as vitellogenesis, which is regulated by the production of 20E shortly after a blood meal (7, 19, 53). In addition, the male transfer of 20E during mating further contributes to oogenesis to maximize female fecundity (15–17). While genetic approaches that ablate mosquito ovary development have identified an essential role of 20E signaling to establish *Plasmodium* infection in the mosquito host (21, 33), the application of a 20E agonist provides contrasting evidence that boosting 20E signaling can also impair malaria parasite infection (20). These data argue that distinct mechanisms of 20E signaling may impair malaria parasite survival in the mosquito host through its influence on mosquito reproduction or the innate immune system. However, studies of 20E function on the immune system of mosquitoes and other insects have thus far been limited.

**FIG 5:**
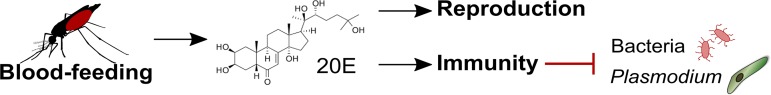
Multimodal effects of blood feeding and 20E on mosquito reproduction and immunity. Blood feeding stimulates the production of 20E, which can initiate vitellogenesis and influence mosquito fecundity as well as prime mosquito immune responses that limit bacterial and malaria parasite survival.

Much of our current understanding of 20E function in innate immunity relies on previous studies in *Drosophila* (23–26). 20E signaling regulates antimicrobial protein (AMP) production in *Drosophila* (23, 24) through PGRP-LC-dependent and -independent mechanisms via the ecdysone receptor and ultraspiracle heterodimer (24). Evidence from *Drosophila* also suggests that 20E promotes hemocyte activation, leading to increased mobility, responsiveness to wounding, and phagocytic activity (26, 54). Through the results presented here, we see similarities in which 20E regulates AMP production (cecropins) and increases the phagocytic activity of mosquito immune cells. We also find that 20E mediates antipathogen effects on both bacteria and malaria parasites (Fig. 5), similar to the antibacterial effects of 20E signaling described in *Drosophila* (24, 26). Despite these parallels, there are distinct physiological differences between *Drosophila* and *An. gambiae*, most notably in the blood-feeding behavior of mosquitoes, pathogen exposure, and the production of 20E. For this reason, there is reason to believe that the role of 20E may vary between *Drosophila* and mosquitoes, particularly regarding innate immunity.

In mosquitoes, several studies suggest that blood feeding, independent of pathogen challenge, stimulates mosquito immunity (28, 30–32). However, these studies only indirectly implicate the role of 20E function in the mosquito immune response. More direct evidence demonstrates the role of 20E in prophenoloxidase (PPO) expression *in vitro* (27) and that of LRIM9, a leucine-rich immunomodulatory protein, through experiments *in vivo* (28). Previous studies show that both LRIM9 (28) and multiple PPOs (29) influence *Plasmodium* killing, supporting a model in which 20E promotes an anticipatory immune response to immune challenge immediately following a blood meal (28). When combined with previously described antibacterial and anti-*Plasmodium* responses of other immune genes identified in our RNA-seq analysis (43–47), our experiments demonstrate the activation of mosquito innate immunity by 20E.

Given the role of 20E priming in bacteria and malaria parasites, 20E likely triggers both cellular and humoral immune components that limit pathogen survival. We demonstrate that 20E increases the phagocytic activity of mosquito immune cells and the production of AMPs, as well as other immune elicitors. However, the mechanisms that establish 20E-mediated immune priming remain unknown. Following 20E treatment, we see distinct changes in cellular immune function, yet it is unclear if 20E also influences humoral components produced by hemocytes or the fat body that are secreted into the hemolymph that may also act on bacteria or invading malaria parasites. It is also possible that the introduction of bacteria into the hemolymph and the invasion of *Plasmodium* via an infected blood meal may invoke distinct 20E-mediated immune responses, thus requiring further study.

Through our challenge experiments, we observed temporal differences in the effects of 20E priming. E. coli challenge experiments appeared more transient, displaying peak activity 12 to 18 h postpriming, contrasting with P. berghei challenge experiments that retain activity ∼48 h postpriming that persists through the onset of ookinete invasion. In both approaches, we believe that the injection of 20E likely creates an initial priming signal. Yet, the more persistent priming that accompanies the P. berghei infection experiments may be explained by the second pulse of endogenous 20E that accompanies the infected blood meal to provide a continued boost to 20E-mediated immunity.

In addition to the impacts of 20E on mosquito immunity, our RNA-seq analysis identified differentially regulated genes that mediate a wide array of physiological responses to 20E treatment. This includes genes involved in lipid metabolism and transport that likely contribute to vitellogenesis and the production of other lipid resources synthesized shortly after a blood meal. Since these lipids can be incorporated into egg production or developing oocysts (21, 55, 56), the influence of 20E on immunity and physiology raise additional considerations concerning the potential trade-offs between mosquito reproduction and immunity. Both physiological processes are energetically costly, where 20E production may serve as a limiting factor for the allocation of resources toward egg production or immunity (57). Evidence suggests that mosquito fitness is reduced at the cost of anti-*Plasmodium* immunity (55, 58), arguing that there is a competition for resources in the mosquito host. This is supported by fitness experiments in transgenic mosquitoes refractory to malaria infection that display increased fecundity following *Plasmodium* challenge, presumably by enabling more resources for egg production (59, 60). However, this was challenged by recent studies arguing that Plasmodium falciparum infection intensity positively correlates with increased egg production and levels of 20E, where the production of lipids during vitellogenesis is used by P. falciparum to increase survival and optimize transmission (21). Therefore, the effects of 20E on mosquito immunity and reproductive fitness potentially depend on specific host-pathogen interactions that define differences in *An. gambiae* immunity to P. berghei and P. falciparum parasites (45, 61).

In summary, our findings demonstrate the role of the hormone 20E in priming mosquito innate immunity to both bacteria and malaria parasites. These effects are mediated in part through the activation of cellular immunity and likely involve a number of humoral factors, including that of CEC3 described here, that make mosquitoes more resistant to pathogen infection. As a result, these data provide novel insights into the hormonal regulation of the mosquito immune system yet require further investigation to better understand the regulation and tissue-specific contributions of 20E immune priming. Together with previous work (20), our results support that increasing 20E signaling in the mosquito host can reduce *Plasmodium* infection and may serve as a potential target to interrupt the transmission of malaria.

## MATERIALS AND METHODS

### Ethics statement.

The protocols and procedures used in this study were approved by the Animal Care and Use Committee at Iowa State University (IACUC-18-228).

### Mosquito rearing and *Plasmodium* infections.

A colony of *An. gambiae* G3 was maintained at 27°C and 80% relative humidity, with a 14-h/10-h light/dark cycle. Mosquito larvae were fed on a ground fish food diet (Tetramin). Pupae were isolated using a pupal separator (John W. Hock Company) and were allowed to emerge in containers of ∼50 mosquitoes. Adults eclosed in mixed populations of male and female mosquitoes where mating likely occurred prior to downstream experimentation but was not quantified. Adult mosquitoes were maintained on a 10% sucrose solution.

For mosquito infections, an mCherry strain of Plasmodium berghei (62) was passaged into female Swiss Webster mice and monitored for exflagellation as previously described (29, 36, 63). Mosquitoes were fed on an infected, anesthetized mouse, and then maintained at 19°C. Mosquito midguts were dissected 8 days postinfection in 1× phosphate-buffered saline (PBS), and oocyst numbers were measured by fluorescence using a Nikon Eclipse 50i microscope.

### RNA-seq analysis.

To examine the effects of 20E on mosquito cells, we applied 20E (dissolved in 100% ethyl alcohol [EtOH]) to Sua 4.0 cells to a final concentration of 500 ng/ml (1 μM) based on comparable studies in *Drosophila* (24) and *An. gambiae* cell lines (27) that used the same concentration. Sua 4.0 cells treated with a comparable volume of 100% EtOH were used as controls. Twenty-four hours after application, Sua 4.0 cells for both 20E-treated and control samples were harvested for RNA isolation. Total RNA was isolated using TRIzol (Thermo Fisher Scientific) and then purified with the RNA Clean & Concentrator-5 kit (Zymo Research) and quantified using a Nanodrop spectrophotometer (Thermo Fisher Scientific). RNA quality and integrity were measured using an Agilent 2100 Bioanalyzer Nano Chip (Agilent Technologies). Two hundred nanograms of total RNA was used to perform RNA-seq analysis from four independent biological replicates. RNA-seq libraries were prepared by the Iowa State University DNA Facility using the TruSeq stranded mRNA sample prep kit (Illumina) and dual indexing according to the manufacturer’s instructions. The size, quality, and concentration of the libraries were measured using an Agilent 2100 Bioanalyzer and a Qubit 4 fluorometer (Invitrogen), and the libraries were then diluted to 2 nM based on the size and concentration of the stock libraries. Clustering of the libraries into a single lane of the flow cell was performed with an Illumina cBot. Paired-end sequencing of 150 bp was performed on an Illumina HiSeq 3000 using standard protocols.

Raw sequencing data were analyzed by the Iowa State Genome Informatics Facility. Sequence quality was assessed using FastQC (v 0.11.5) (64), and then paired-end reads were mapped to the Anopheles gambiae PEST reference genome (AgamP4.9) downloaded from VectorBase (65) using STAR aligner (v 2.5.2b) (66). Genome indexing was performed using the genomeGenerate option and the corresponding GTF file downloaded from VectorBase (version 4.7), followed by mapping using the alignReads option. Output SAM files were sorted and converted to BAM format using SAMTools (v 1.3.1) (67), and counts for each gene feature were determined from these alignment files using featureCounts (v 1.5.1) (68). Reads that were multimapped or chimeric and fragments with missing ends were excluded. Counts for each sample were merged using AWK script, and differential gene expression analyses were performed using edgeR (69). Differentially expressed genes with a *q* score of ≤ 0.1 were considered significant and were used for downstream analyses.

### *In vivo* injection of 20E in mosquitoes.

Adult female *An. gambiae* mosquitoes (3 to 5 days posteclosion) were injected with 20E essentially as described by Upton et al. (28). Briefly, 20E (Sigma) was resuspended in 100% EtOH and diluted (1:10) in 1× PBS to a working stock suspension of 7.24 μg/μl. Anesthetized mosquitoes were injected with 69 nl of the working stock solution, resulting in the delivery of 500 ng of 20E per individual mosquito, an amount half that of previous experiments (28). This dosage was previously shown to maximally stimulate protein synthesis in the fat body of Aedes aegypti mosquitoes *in vitro* (28, 70). Control mosquitoes were similarly injected with 69 nl of 100% EtOH diluted (1:10) in 1× PBS. After injection, surviving mosquitoes were challenged (E. coli or P. berghei) or were used for the collection of samples for RNA isolation to determine the effects of 20E on gene expression. Mosquitoes were kept at 19°C for bacterial challenge and P. berghei infection experiments to avoid confounding effects of temperature in assessing the role of 20E in immune function.

### Gene expression analysis.

Total RNA was isolated from Sua 4.0 cells or whole mosquitoes (∼10 mosquitoes) using TRIzol (Thermo Fisher Scientific) according to the manufacturer’s protocol. RNA samples were quantified using a NanoDrop spectrophotometer (Thermo Fisher Scientific), and ∼2 μg of total RNA was used as a template for cDNA synthesis using the RevertAid first-strand cDNA synthesis kit (Thermo Fisher). Gene expression was measured by qRT-PCR using gene-specific primers (see Table S1 in the supplemental material) and PowerSYBR Green (Invitrogen). The results were normalized to an S7 reference gene and quantified using the 2^−ΔΔ^*^CT^* method as previously described (71). Samples were run in triplicate for each experiment.

### dsRNA synthesis and gene silencing.

T7 primers for green fluorescent protein (GFP) and cecropin 3 (*cec3*) were used to amplify cDNA prepared from whole *An. gambiae* mosquitoes and cloned into a pJET1.2 vector using a CloneJET PCR cloning kit (Thermo Fisher). The resulting plasmids were used as a template for amplification using the corresponding T7 primers (Table S2). PCR products were purified using the DNA Clean & Concentrator kit (Zymo Research) and used as a template for double-stranded RNA (dsRNA) synthesis as previously described (36, 63). The resulting dsRNA was resuspended in RNase-free water to a concentration of 3 μg/μl. For gene silencing experiments, 69 nl of dsRNA was injected per mosquito and evaluated by qRT-PCR to establish gene knockdowns at 1 to 5 days postinjection. Time points with the highest efficiency of gene silencing were chosen for downstream experiments.

10.1128/mSphere.00983-19.4 TABLE S2 Primers used for dsRNA synthesis. Download Table S2, PDF file, 0.04 MB. Copyright © 2020 Reynolds et al. 2020 Reynolds et al. This content is distributed under the terms of the Creative Commons Attribution 4.0 International license.

10.1128/mSphere.00983-19.5 TABLE S3 Differentially regulated genes in Sua 4.0 cells following 20E treatment. Download Table S3, XLSX file, 0.02 MB. Copyright © 2020 Reynolds et al. 2020 Reynolds et al. This content is distributed under the terms of the Creative Commons Attribution 4.0 International license.

### Phagocytosis assays.

*In vivo* phagocytosis assays were performed as previously described (29, 36). Briefly, mosquitoes were injected with 69 nl of red fluorescent FluoSpheres (1 μm; Molecular Probes) at a 1:10 dilution in 1× PBS. Following injection, mosquitoes were allowed to recover for 2 h at 19°C and were then perfused onto a multitest slide. Samples were fixed using 4% paraformaldehyde for 30 min, washed three times using 1× PBS, and then blocked in 1% bovine serum albumin (BSA) for 30 min at room temperature. Hemocytes were stained using a 1:500 dilution of fluorescein isothiocyanate (FITC)-labeled wheat germ agglutinin (WGA; Sigma) in 1× PBS and incubated overnight at 4°C. After washing with 1× PBS, cell nuclei were stained with ProLong Gold antifade reagent with DAPI (4′,6-diamidino-2-phenylindole) (Invitrogen). Hemocytes were identified by the presence of WGA and DAPI signals, and the percent phagocytosis was calculated by dividing the number of cells containing red fluorescent beads by the total number of cells present. The phagocytic index was calculated by counting the total number of beads per cell (this is summed for all of the cells) and dividing that number by the number of phagocytic cells. Approximately 200 cells were counted per mosquito sample.

### E. coli challenge.

To evaluate the effect of blood feeding and 20E priming on bacterial infection, bacterial challenge experiments were performed as previously described (72) with control or 20E-treated mosquitoes. Briefly, kanamycin-resistant E. coli was cultured at 37°C until reaching an optical density at 600 nm (OD_600_) of 0.4. Approximately 1 ml of the E. coli solution was centrifuged at 10,000 × *g* for 10 min, and the supernatant was removed. The pellet was washed twice with 1× PBS and then concentrated, before resuspension in 1× PBS. Challenge experiments were performed by injecting 69 nl of E. coli into the thorax of each mosquito under each experimental condition. After injection, E. coli-challenged mosquitoes were kept at 19°C for 12 to 24 h before perfusion. The hemolymph from five mosquitoes (∼50 μl) was pooled and diluted in 450 μl of LB broth at room temperature. One hundred microliters of the pooled hemolymph sample was plated on LB agar plates containing kanamycin in triplicate and incubated overnight at 37°C. The number of E. coli colonies per plate was recorded, with the average used for a comparison between control and 20E-treated samples (number of E. coli colonies/average of control). This standardization was performed to normalize for variation in E. coli numbers between experiments.

To determine the effects of *cec3* gene silencing on bacterial load, a slightly modified procedure was used to evaluate individual mosquito bacterial titers to account for additional variability in individual gene-silenced mosquitoes (48, 73). Briefly, control and *cec3*-silenced mosquitoes were challenged 2 days post-dsRNA injection with 69 nl of kanamycin-resistant E. coli, as described above. Twenty-four hours post-E. coli challenge, individual mosquitoes were homogenized in 100 μl of 1× PBS. Samples were then diluted with an additional 100 μl of 1× PBS and vortexed, and 50 μl of each sample was plated on kanamycin agar plates. Plates were incubated overnight at 37°C, and the number of colonies was counted and converted to CFU/ml.

### Data availability.

Gene expression data were deposited in the NCBI Gene Expression Omnibus (74) and are accessible under GEO accession number GSE116252.
