# Supplementary figures and images for: 20-Hydroxyecdysone Primes Innate Immune Responses That Limit Bacterial and Malarial Parasite Survival in Anopheles gambiae
Source: mSphere. 2020 Apr 15;5(2):e00983-19. doi: 10.1128/mSphere.00983-19 (PMC7160685; doi:10.1128/mSphere.00983-19)

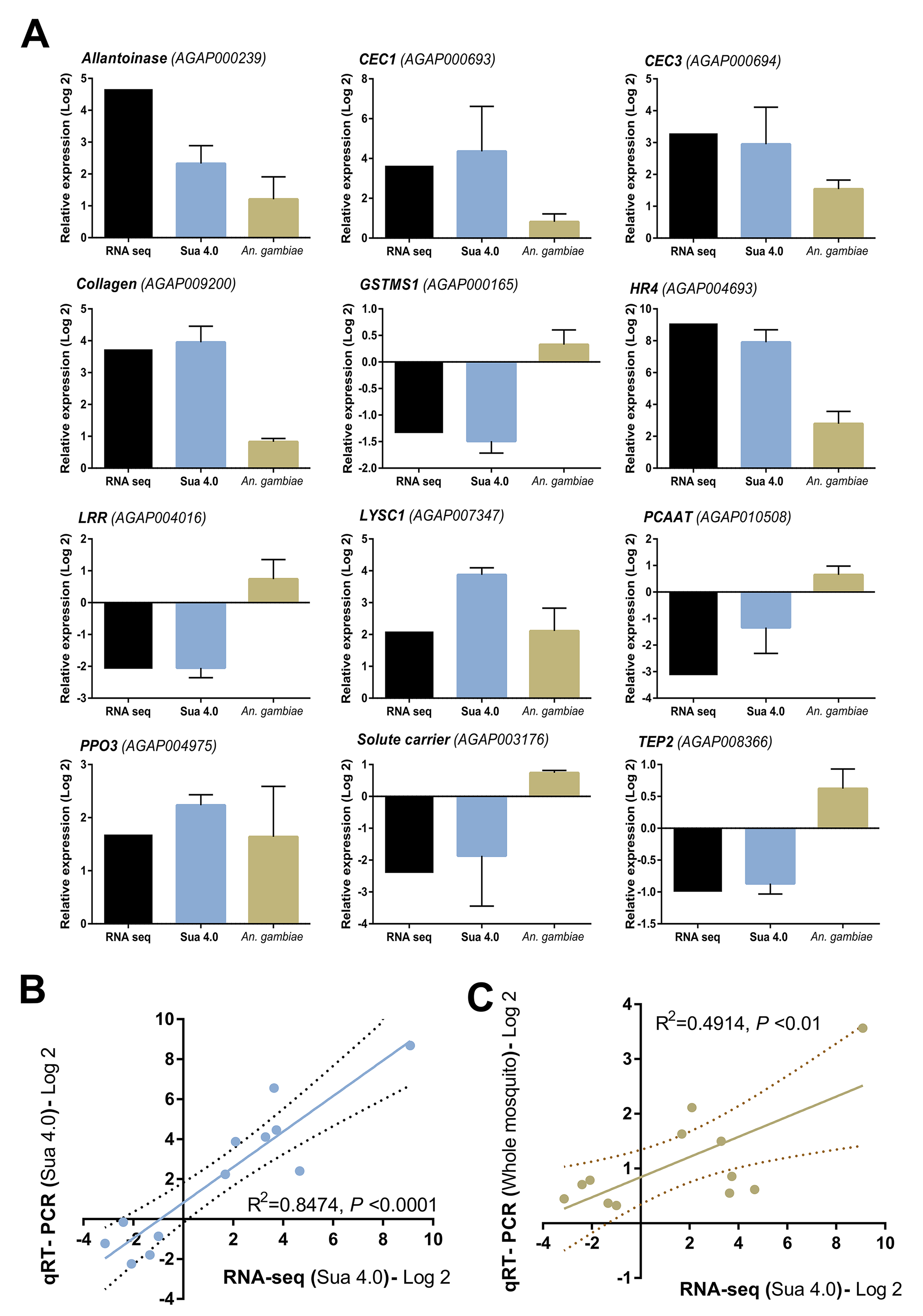

Supplement: FIG S1 [file mSphere.00983-19-sf001.tif]

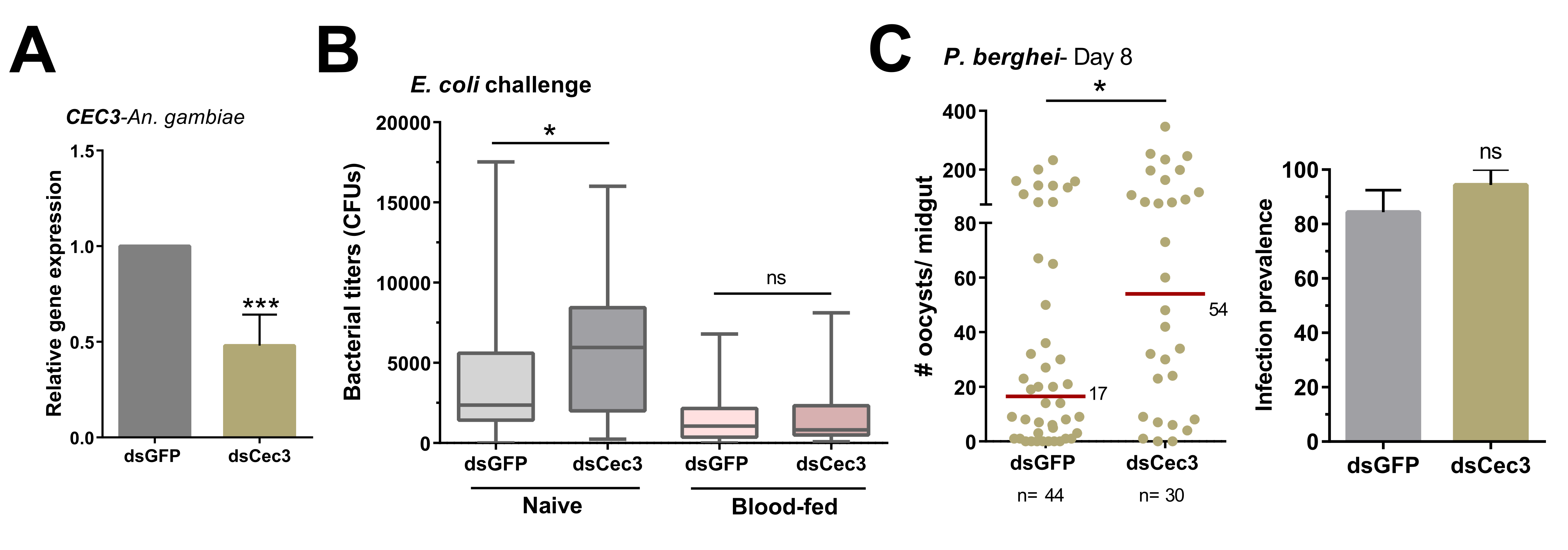

Supplement: FIG S2 [file mSphere.00983-19-sf002.tif]
