# Supplementary material for: 20-Hydroxyecdysone Primes Innate Immune Responses That Limit Bacterial and Malarial Parasite Survival in Anopheles gambiae
Source: mSphere. 2020 Apr 15;5(2):e00983-19. doi: 10.1128/mSphere.00983-19 (PMC7160685; doi:10.1128/mSphere.00983-19)
Supplement: TABLE S1 [file mSphere.00983-19-st001.pdf]

Table S1. Primers used for qRT-PCR analysis

Primer	Gene ID	Sequence (5'- 3')
Allantoinase-F	AGAP000239	CCCACGACGGTAAGGTGACG
Allantoinase-R		TCCCGGCATGAGCATCAAAT
Cecropin 1-F	AGAP000693	TTCATCTTTGTCGTGCTGGC
Cecropin 1-R		GCACTGCCAGCACGACAAAG
Cecropin 3-F	AGAP000694	ACGTACTGAACCACCTGCGCGTT
Cecropin 3-R		GCGCTGTGTGCGCCGATGAA
Collagen-F	AGAP009200	TGGGACTGCGAGGATTCGAG
Collagen-R		ACCTCGTCCGACTGGCTGTG
GTMS1-F	AGAP000165	GCGGTGCTGGTGGTGAAGAT
GTMS1-R		CGTCCGGATCGTCGAACTTG
HR4-F	AGAP004693	TCGGGGTCAAATGCATCACA
HR4-R		GCTGCAGTTGGGTTCCGAGA
LRR-F	AGAP004016	GCTCGTTTTGTGCCGGAATG
LRR-R		GCCCGCTTCCTGCAGCTTAT
LYSC1-F	AGAP007347	TTCAGCACATCGGCGACAAA
LYSC1-R		TCCAGCCGTACCAGGCGTTA
PCAAT-F	AGAP010508	TGCGGTGTTCCGGATATTGG
PCAAT-R		AGGCGCCACTTCATCCATCC
PPO3-F	AGAP004975	CTATTCGCCATGATCTCCAACTACG
PPO3-R		ATGACAGTGTTGGTTGGTGAAACGGATCT
rpS7-F	AGAP010592	ACCCCATCGAACACAAAGTTGACACT
rpS7-R		CTCCGATCTTTCACATTCCAGTAGCAC
Solute Carrier-F	AGAP003176	GTGCTTGGCTGTGTGCTGGA
Solute Carrier-R		CGTTGGCCTGTACCGTCTCG
TEP2-F	AGAP008366	GCACCTGGCTGACAGCGTTT
TEP2-R		CCCTGACCCTGCACCTCCTT
