# Supplementary material for: 20-Hydroxyecdysone Primes Innate Immune Responses That Limit Bacterial and Malarial Parasite Survival in Anopheles gambiae
Source: mSphere. 2020 Apr 15;5(2):e00983-19. doi: 10.1128/mSphere.00983-19 (PMC7160685; doi:10.1128/mSphere.00983-19)
Supplement: TABLE S2 [file mSphere.00983-19-st002.pdf]

Table S2. Primers used for dsRNA synthesis

Primer	Gene ID	Sequence (5'- 3')
GFP-T7-F		TAATACGACTCACTATAGGGAGAATGGTGAGCAAGGGCGAGGAGCTGT
GFP-T7-R		TAATACGACTCACTATAGGGAGATTACTTGTACAGCTCGTCCATGCC
Cecropin 3-T7-F	AGAP000694	TAATACGACTCACTATAGGGTCAGTCTGAGATCTCTTCCCGT
Cecropin 3-T7-R		TAATACGACTCACTATAGGGAGATTACTTGTACAGCTCGTCCATGCC
